# Artificial Intelligence as a Potential Catalyst to a More Equitable Cancer Care

**DOI:** 10.2196/57276

**Published:** 2024-08-12

**Authors:** Sebastian Garcia-Saiso, Myrna Marti, Karina Pesce, Silvana Luciani, Oscar Mujica, Anselm Hennis, Marcelo D'Agostino

**Affiliations:** 1 Pan American Health Organization Washington, DC United States; 2 Hospital Italiano de Buenos Aires Buenos Aires Argentina

**Keywords:** digital health, public health, cancer, artificial intelligence, AI, catalyst, cancer care, cost, costs, demographic, epidemiological, change, changes, healthcare, equality, health system, mHealth, mobile health

## Abstract

As we enter the era of digital interdependence, artificial intelligence (AI) emerges as a key instrument to transform health care and address disparities and barriers in access to services. This viewpoint explores AI's potential to reduce inequalities in cancer care by improving diagnostic accuracy, optimizing resource allocation, and expanding access to medical care, especially in underserved communities. Despite persistent barriers, such as socioeconomic and geographical disparities, AI can significantly improve health care delivery. Key applications include AI-driven health equity monitoring, predictive analytics, mental health support, and personalized medicine. This viewpoint highlights the need for inclusive development practices and ethical considerations to ensure diverse data representation and equitable access. Emphasizing the role of AI in cancer care, especially in low- and middle-income countries, we underscore the importance of collaborative and multidisciplinary efforts to integrate AI effectively and ethically into health systems. This call to action highlights the need for further research on user experiences and the unique social, cultural, and political barriers to AI implementation in cancer care.

## Introduction

In an era called the Age of Digital Interdependence by the United Nations Secretary-General [1], where technological advancements continually reshape the world, the health sector is facing a significant transformation.

Artificial Intelligence (AI) emerges not just as a technological innovation but as a critical instrument with the potential to help overcome critical health challenges, including health care costs, unmet health needs related to the double burden of infectious and noncommunicable diseases, a considerable shortage of trained health professionals, and more importantly, the profound and long-standing inequities in the distribution of the opportunities to health care and well-being [2].

This viewpoint explores the potential of AI as a catalyst in bridging the gap in cancer health care, from a broader and scientifically grounded perspective on its integration into health systems. Fulfilling health equity, however, goes beyond achieving digital equity: leaving no one behind in the digital age requires not only reaching those who are not digitally literate but also populations in situations of greatest social, economic, geographic, and cultural vulnerability or disadvantage—the proverbial determinants of, and barriers to, timely and quality access to health care. Information and communications technologies have the potential to reduce health inequalities by enabling people to access information and digital tools for prevention and care at the right time and in the right format. Digital inclusion involves ensuring appropriate access, digital skills, usability, and navigability in the development of technological solutions. This approach should promote inclusion while respecting the autonomy of individuals and groups who decide not to use digital services [3,4].

## The Current Landscape

Cancer continues to pose a great and ever-growing burden of disease, being the second leading cause of death worldwide. Recently released estimates from the World Health Organization account for nearly 20 million incident cases and 10 million deaths in 2022, projecting almost double these figures by the year 2050 [5]. Although the cumulative risk of developing cancer before the age of 75 years is unequivocally greater in countries with a higher Human Development Index and larger income per capita, most of the cancer burden is concentrated in countries with lower Human Development Index and smaller income per capita. More dramatically, inequalities in cancer care—as is usually the case with noncancer burden as well—bear a disproportionate impact in underserved populations [6]. These inequalities, shaped by highly context-specific socioeconomic, geographical, and cultural barriers, manifest in varied health care dimensions—from access to screening to palliative care, diagnostic accuracy, treatment options, pain management, premature mortality, survival, quality of life, and other health outcomes. This web of challenges underscores the need for a comprehensive and customized approach to equitable access to health, one that is robustly supported by the digital transformation of the health sector [7]. In this context, AI presents a unique opportunity to facilitate and improve access to health services, making it more equitable, accessible, and personalized, especially for underserved communities.

## AI as a Transformative Agent in Reducing Health Care Inequalities

**Figure 1:**
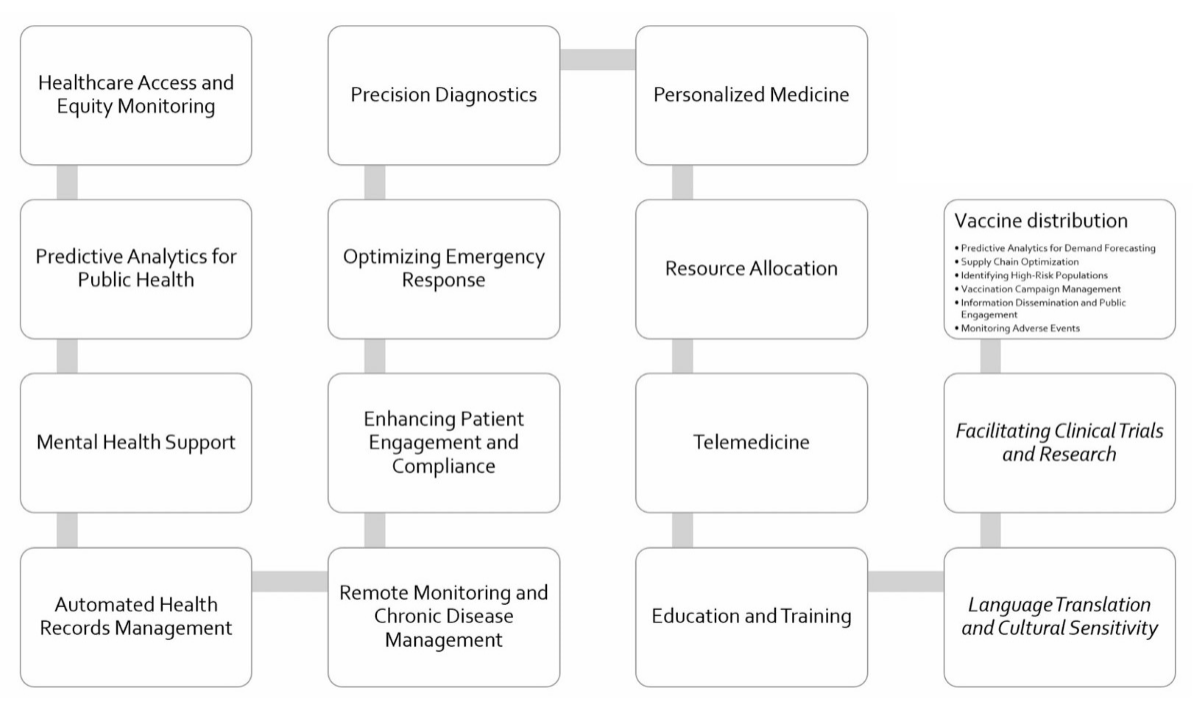
Artificial intelligence (AI) as a transformative agent in reducing health care inequalities.

AI’s capacity to process and analyze vast amounts of data swiftly and accurately positions it as an invaluable tool in health care. Its applications range from enhancing diagnostic precision to optimizing resource allocation and extending health care reach to remote areas [8]. The implications of these advancements are profound, especially in regions where health care resources are scarce, and the burden of disease is high. AI can play a significant role in making health care more inclusive, accessible, and effective for all segments of the population, particularly in the following areas (Figure 1):

### Health Care Access and Equity Monitoring

AI can analyze health service utilization patterns to identify inequalities in access to care. This information can inform policies and strategies to make health care more equitable. AI algorithms evaluate data on health care use, patient demographics, and service availability. This analysis identifies regions or groups with reduced access, guiding targeted policy interventions to address these gaps [9].

### Predictive Analytics for Public Health

AI can analyze data from various sources to predict outbreaks and public health emergencies. This foresight can help in deploying resources more effectively to underserved areas, potentially preventing or mitigating health crises. By aggregating and analyzing health care data, social media, environmental conditions, and other relevant sources, AI models can forecast potential health threats, allowing for proactive resource allocation and emergency planning in vulnerable areas [10,11].

### Mental Health Support

AI-driven chatbots and virtual assistants can provide preliminary mental health support, especially in regions lacking mental health professionals. These tools can offer coping strategies, guidance, and early intervention. Utilizing natural language processing and sentiment analysis, AI tools interact with users, offering support, and identifying those who may require urgent care, thereby bridging the gap in mental health services [12,13].

### Automated Health Records Management

AI can streamline health records management, ensuring that patient data are accurately recorded and easily accessible. This efficiency can improve health care delivery, particularly in underresourced settings. AI systems automate the sorting, storage, and retrieval of electronic health records, reducing errors and saving time. This enhancement in overall health care efficiency is especially critical in underresourced areas [14,15].

### Remote Monitoring and Chronic Disease Management

AI can be used in remote patient monitoring systems to track the health status of patients with chronic diseases, alerting health care providers to changes that may require intervention. AI algorithms analyze data from wearable devices and home monitoring equipment, detecting deviations in health metrics, enabling timely interventions and better management of chronic conditions [8,16].

### Enhancing Patient Engagement and Compliance

AI-powered applications can remind patients about medication schedules, appointments, and health check-ups, especially helping those with limited access to regular health care. These AI tools personalize reminders and health tips based on patient data and interaction patterns, increasing adherence to treatment and preventive care routines [17-19].

### Optimizing Emergency Response

AI algorithms can help in planning and optimizing emergency medical responses, ensuring quicker and more efficient care delivery during critical situations. By simulating various emergency scenarios and analyzing historical response data, AI can optimize resource allocation, route planning for ambulances, and emergency room preparedness, enhancing the responsiveness of emergency services [20].

### Precision Diagnostics

AI algorithms can rapidly analyze complex medical data, leading to more accurate diagnoses and early intervention, especially in areas lacking specialist health care providers. AI models, particularly those trained in image recognition and pattern detection, can assist in diagnosing diseases from medical imagery and lab results with high accuracy, supplementing the expertise in underserved areas [21].

### Personalized Medicine

By considering individual genetic, environmental, and lifestyle factors, AI can tailor treatment plans, ensuring each patient receives the most effective care. AI systems analyze patient-specific data, including genetic profiles and health histories, to predict individual responses to different medical treatments, enabling more effective, customized care [22,23].

### Resource Allocation

In resource-limited settings, AI can optimize the use of medical supplies and personnel, ensuring the most efficient use of available resources. AI tools forecast health care demands and optimize the distribution and allocation of medical resources, helping to ensure that scarce resources are used where they are most needed.

### Telemedicine

AI-enhanced telemedicine can bridge distances, bringing expert medical advice to the most remote corners of the globe. AI supports telemedicine through diagnostic assistance, patient management systems, and enhanced communication tools, making health care accessible in remote and underserved areas.

### Education and Training

AI’s role in educating health care professionals is invaluable, providing access to the latest medical knowledge and training, irrespective of geographical barriers. AI-driven educational platforms and simulations adapt to individual learning styles and needs, offering health care professionals personalized and up-to-date medical training.

### Language Translation and Cultural Sensitivity

AI-powered tools can provide real-time translation services, making health care more accessible to nonnative speakers and reducing cultural barriers. These tools can also be trained to recognize and adapt to cultural nuances in patient care. AI-based translation and cultural sensitivity tools analyze and adapt health care information and interactions to various languages and cultural contexts, thereby enhancing the accessibility and effectiveness of health care services for diverse patient groups.

### Facilitating Clinical Trials and Research

AI can assist in identifying suitable candidates for clinical trials, particularly from underrepresented groups, ensuring broader inclusivity in research. AI algorithms analyze vast amounts of health care data to identify potential clinical trial participants, considering various factors like genetic profiles, health conditions, and demographic characteristics. This process helps in creating more diverse and representative participant groups for clinical trials, which is essential for the generalizability and effectiveness of medical research [24,25].

### Vaccine Distribution

AI is also revolutionizing the approach to vaccine distribution and management, a crucial aspect of health equity, particularly in the context of pandemics. AI tools and algorithms can optimize vaccine distribution strategies, ensuring that vaccines are delivered efficiently and equitably. AI offers a range of solutions, from predictive analytics for demand forecasting to supply chain optimization, each addressing a key facet of the vaccine distribution challenge. These AI-driven approaches are not only enhancing the effectiveness of vaccination campaigns but also ensuring that high-risk populations are prioritized and that public health messages are communicated effectively.

## Discussion

### AI’s Role in Ensuring Equitable Cancer Care

The rapid advancement of AI in health care, particularly in the fight against cancer, has sparked new expectations about its capabilities. However, it is crucial to critically examine AI’s role in ensuring equitable cancer care. This discussion emphasizes the necessity for AI models to incorporate globally diverse data, highlighting the need for inclusive development practices and an ethical commitment to making these potentially life-saving technologies accessible to all, not just a select few. It underscores the importance of considering a wide range of genetic and environmental factors, as well as the need for data that universally represents diverse populations. These considerations form a compelling case for a holistic and fair approach. As we progress in applying AI to cancer research, we must also strive to realize a future where advanced cancer treatments are both effective and equitable for every individual, irrespective of their geographic location or socioeconomic status. Some particular reflections are highlighted below.

### Equity in AI Development: Serving All Communities in the Technological Era

It is essential that emerging technologies are not shaped only by technology companies and those in wealthy countries. If models are not trained on data from people in underresourced places, those populations might be poorly served by the algorithms. AI development must prioritize inclusivity, ensuring that datasets reflect the global population's diversity. This inclusivity extends beyond data collection to involve collaboration with local health care providers and communities to understand and address unique health care challenges. The integration of diverse data sources can improve the robustness and accuracy of AI models, leading to more effective and equitable health care solutions [26,27].

### Global Perspectives in AI: Bridging the Gap in Cancer Care

It is imperative that AI technologies are developed with a global perspective. Ensuring that AI models incorporate diverse datasets, including those from less affluent regions, is not just an ethical imperative but necessary for providing equitable health care outcomes for all. Global collaboration in AI research and development can bridge the gap in cancer care by sharing knowledge, resources, and best practices across borders. Such efforts can lead to AI tools that are adaptable to various health care settings and capable of addressing the unique needs of different populations [28,29].

### Data Diversity: The Key to Personalized AI in Oncology

For AI to truly be a force for good in cancer treatment, we must broaden the scope of data collection to encompass the varied genetic and environmental factors present in all communities, offering precise and personalized care to every patient regardless of their geographic or socioeconomic factor. Diverse data sources, including genomic data, clinical records, and environmental exposure information, can enhance AI’s ability to identify risk factors, predict disease progression, and tailor treatments to individual patients. This approach ensures that AI-driven cancer care is not only precise but also personalized, improving outcomes for patients worldwide [30].

### Mitigating Bias in AI Algorithms

Bias in AI algorithms can significantly affect health care outcomes, particularly for underserved and marginalized communities. Addressing this issue requires comprehensive strategies to ensure AI systems are equitable and devoid of discriminatory biases. One critical approach is to use diverse and representative datasets, actively collecting data from underrepresented groups to create inclusive AI models. Additionally, implementing robust bias detection and correction methods, such as fairness-aware machine learning, helps identify and rectify biases in data and algorithms. Enhancing transparency and explainability allows stakeholders to understand AI decision-making processes, ensuring greater accountability. Inclusive development practices, involving ethicists, sociologists, and representatives from marginalized communities, provide valuable insights to address potential biases. Establishing comprehensive ethical guidelines and frameworks is essential to address issues like data privacy, informed consent, algorithmic transparency, and accountability [31,32].

### Diversifying AI Learning to Combat Health Care Inequalities in Cancer

The effectiveness of AI in cancer care hinges on the diversity of its learning. Without incorporating data from underrepresented groups, we risk perpetuating existing health care disparities. Therefore, it is our collective responsibility to ensure that AI systems are as diverse as the populations they aim to serve [33].

### Cultural Convergence and the Use of AI in Cancer Treatments

It is essential to consider cultural differences, which could, in some cases, be subtle but significant, as they influence the manifestation of the disease, responses to treatment, and patient care preferences. AI models must not only be trained on diverse data sets but must also be sensitive to the cultural contexts that shape health behaviors and outcomes. This approach reinforces the need for multifaceted equity that goes beyond data diversity to encompass the entire human experience in cancer treatment. Culturally sensitive AI models can improve patient engagement, adherence to treatment protocols, and overall satisfaction with care, leading to better health outcomes [34,35].

### Telemedicine and Remote Monitoring

AI-enhanced telemedicine can bridge distances, bringing expert medical advice to the most remote corners of the globe. AI supports telemedicine through diagnostic assistance, patient management systems, and enhanced communication tools, making health care accessible in remote and underserved areas. Remote monitoring systems powered by AI can track the health status of patients with chronic diseases, alerting health care providers to changes that may require intervention. This continuous monitoring can prevent complications and reduce the need for hospital visits, thus alleviating the burden on health care systems and improving patients' quality of life [8,16].

### Education and Training

AI’s role in educating health care professionals is invaluable for all areas, providing open and real-time access to the latest medical knowledge, training materials, and learning objects, irrespective of geographical barriers. AI-driven educational and simulation platforms adapt to individual learning styles and needs, offering health care professionals personalized and up-to-date medical training. This approach ensures that all health care workers, regardless of their location, have access to the best practices and emerging knowledge in cancer care, enhancing the overall quality of care provided to patients [36].

### Resource Allocation and Optimization

AI can play a critical role in optimizing resource allocation in health care settings, particularly in underresourced regions. Through the examination of patterns in health care utilization, AI can identify areas where resources are most needed and predict future demands. This capability is crucial for efficient health care delivery, ensuring that medical supplies, personnel, and infrastructure are utilized optimally. For example, AI algorithms can help in planning and optimizing emergency medical responses, ensuring quicker and more efficient care delivery during critical situations. This optimization can significantly enhance the responsiveness of health care systems, especially in emergencies, ultimately improving patient outcomes [20].

By analogy with precision medicine, precision public health has been conceptualized as the practice that aims at multidimensionally characterizing social position and accurately pinpointing mechanisms to reduce health inequities [37]. AI can play a crucial role in providing ever greater precision in public health, inasmuch as it directs its prodigious capabilities toward ensuring their equitable distribution across populations and territories. In the context of exceedingly unequal societies with highly segmented and fragmented health care systems, under the rule of the inverse health equity law [38], it is worth recalling the four equity considerations for the use of AI in public health, proposed by Smith et al [39] as the starting point for the promotion of equitable AI in public health—the digital divide, algorithmic bias and values, plurality of values across systems, and fair decision-making procedures. It should be quite clear that strengthening equity monitoring is paramount when introducing AI technologies to make sure they do not inadvertently increase or create inequities [40].

### Case Studies of AI Implementation in Health Care

Real-world examples of AI implementation in health care provide concrete evidence of its impact on reducing disparities and offer valuable insights into best practices, challenges encountered, and strategies for overcoming these challenges. One notable example is the use of AI in breast cancer screening in the United States. Researchers developed an AI model that outperformed radiologists in detecting breast cancer from mammograms, demonstrating the potential for AI to enhance diagnostic accuracy and reduce diagnostic disparities [41]. Another significant case is Artemisia, a deep-learning model developed at the Hospital Italiano de Buenos Aires for automatic breast density categorization. Artemisia was validated using 10,229 digital screening mammogram images to classify breast density according to the American College of Radiology’s patterns. This AI system showed significant accuracy, achieving professional-level performance when compared to the majority reports and a commercial software application [42].

### A Call for Collaborative Action

To harness the power of AI in creating a more equitable public health landscape, a concerted, collaborative, and multidisciplinary approach is essential. This initiative calls for a synergy of efforts from various stakeholders in the health care ecosystem. Policy makers, health care providers, AI technology experts, patient advocacy groups, and community leaders all play pivotal roles in shaping how AI is integrated into health care systems. Engaging with policy makers is crucial for establishing regulations and guidelines that ensure the ethical use of AI and addressing critical concerns, such as data privacy, patient consent, and algorithmic transparency. Health care providers, on the front lines of patient care, offer invaluable insights into practical needs and challenges, ensuring that AI solutions are tailored to real-world applications and are patient centric. Collaboration with AI technology experts, including data scientists and engineers, is fundamental for developing robust, accurate, and reliable AI systems. Their expertise is vital in translating health care needs into technological solutions that are both innovative and practical. In addition, it is essential to involve patient advocacy groups and community leaders, particularly from underserved and marginalized populations. Their perspectives and experiences are crucial in identifying and addressing specific health disparities, ensuring that AI solutions are inclusive and equitable. Ethical considerations, such as addressing biases in AI algorithms and ensuring equitable access to AI-enhanced health care, are paramount. Data privacy remains a top concern, especially in handling sensitive health data. Mitigating potential risks, such as unintended consequences of AI decisions or misuse of AI technologies, requires comprehensive strategies and constant vigilance [17].

### The Path Forward

The digital transformation in the health sector is not only about how to use information and communication technologies as supporting tools or about technological modernization alone. Digital transformation is a cultural change that must consider new health care models, process reengineering, systems reorganization, and a deeper understanding of people’s behavior and digital skills. Likewise, such transformation requires a new multisectoral and interdisciplinary approach in the development and implementation of public policies, regulatory frameworks and national digital literacy programs [36].

In this evolving landscape of health care and looking at more resilient health systems, it is crucial to position AI as a critical agent in reducing health disparities. AI’s role should be seen not as replacing human expertise but as augmenting the capabilities of health care professionals, enabling them to deliver more effective and inclusive care. The integration of AI into health care systems is a promising stride toward democratizing health care access and removing barriers related to geography, socioeconomic status, and cultural differences. AI’s potential in identifying and addressing health disparities is profound. Through advanced data analysis and predictive capabilities, AI can illuminate hidden patterns of inequality, guiding targeted interventions where they are most needed. For instance, AI can help tailor public health strategies to address the specific needs of underserved communities, ensuring that preventive care and medical treatments are not only available but also resonate with diverse cultural and socioeconomic contexts. Moreover, AI’s role in enhancing diagnostic accuracy, personalizing treatment plans, and improving patient engagement offers a direct pathway to narrowing the health equity gap. AI, through the timely provision of quality medical care to all persons irrespective of background, works as a key driver toward an equalized health care platform. The adoption of AI in health care is not just a technological shift; it represents a fundamental step toward a health system in which equity and inclusivity are considered key factors for success. AI in cancer treatment offers new opportunities to create a future of health where access to services is possible for every individual who needs it, wherever they need it [43].

### Conclusions

The exploration of AI in cancer care underscores its potential to bridge health care disparities through enhanced diagnostic accuracy, optimized resource allocation, and improved access to care, particularly in underserved communities. Key applications, such as AI-driven health equity monitoring, predictive analytics, mental health support, and personalized medicine, highlight AI’s transformative role. Case studies like the AI model for breast cancer screening in the United States and Artemisia at the Hospital Italiano de Buenos Aires illustrate successful implementations. However, achieving equitable AI integration requires addressing biases, ensuring data diversity, and fostering inclusive development practices. It is crucial to consider local social, cultural, and political contexts to tailor AI solutions effectively. Additionally, more high-quality and disaggregated data at the local level is needed to enhance AI’s accuracy and relevance. Continued research, multidisciplinary collaboration, and ethical considerations are essential for overcoming implementation challenges and maximizing AI’s benefits in cancer care.

## Abbreviations

AI: artificial intelligence
